# Proteomic Analysis of Hippocampus and Cortex in Streptozotocin-Induced Diabetic Model Mice Showing Dementia

**DOI:** 10.1155/2018/8953015

**Published:** 2018-04-05

**Authors:** Kenji Matsuura, Mieko Otani, Masaoki Takano, Keiichi Kadoyama, Shogo Matsuyama

**Affiliations:** ^1^Faculty of Pharmacy, Osaka-Ohtani University, Tondabayashi 584-8540, Japan; ^2^Department of Life Sciences Pharmacy, School of Pharmaceutical Sciences, Kobe Gakuin University, Kobe 650-8586, Japan; ^3^Department of Pharmaceutical Health Care, Faculty of Pharmaceutical Sciences, Himeji Dokkyo University, Himeji 670-8524, Japan; ^4^Biosignal Research Center, Kobe University, Kobe 657-8501, Japan

## Abstract

**Aim:**

Diabetes with its associated hyperglycemia induces various type of peripheral damage and also impairs the central nervous system (CNS). This study is aimed at clarifying the precise mechanism of diabetes-induced dementia as an impairment of CNS.

**Methods:**

The proteomic analysis of the hippocampus and cortex in streptozotocin- (STZ-) treated mouse diabetic model showing dementia was performed using two-dimensional gel electrophoresis (2-DE) followed by mass spectrometry (*n* = 3/group).

**Results:**

Significant changes in the expression of 32 proteins and 7 phosphoproteins were observed in the hippocampus and cortex. These identified proteins and phosphoproteins could be functionally classified as cytoskeletal protein, oxidoreductase, protein deubiquitination, energy metabolism, GTPase activation, heme binding, hydrolase, iron storage, neurotransmitter release, protease inhibitor, transcription, glycolysis, antiapoptosis, calcium ion binding, heme metabolic process, protein degradation, vesicular transport, and unknown in the hippocampus or cortex. Additionally, Western blotting validated the changes in translationally controlled tumor protein, ATP-specific succinyl-CoA synthetase beta subunit, and gamma-enolase isoform 1.

**Conclusions:**

These findings showed that STZ-induced diabetes changed the expression of proteins and phosphoproteins in the hippocampus and cortex. We propose that alterations in expression levels of these proteins play an important role in diabetes-induced dementia.

## 1. Introduction

Diabetes is a chronic and metabolic disease characterized by hyperglycemia resulting from defective insulin secretion and/or insulin resistance [1]. Diabetics are at a significantly elevated risk for nephropathy, peripheral neuropathy, and retinopathy. In the central nervous system (CNS), chronic hyperglycemia leads to the enhanced formation of advanced glycation end products (AGEs), which have potentially toxic effects on neurons, causing dementia [2]. Hyperglycemia also causes a significant increase in generation of reactive oxygen species (ROS), resulting in cerebral angiopathy and abnormalities of neurons and glia in the brain, and subsequent dementia [3]. The degree to which the neuronal abnormality is caused directly by hyperglycemia remains unclear [3]. Moreover, the development of diabetes-induced dementia is not only closely associated with hyperglycemia but also with the action of insulin [4].

Insulin and insulin receptors show abundant expression throughout the brain, especially in the hippocampus, which is involved in dementia [5]. Peripheral insulin crosses the blood-brain barrier (BBB) via an active transport mechanism to exert its effects within the CNS [6]. Insulin receptors in the brain are found at synapses on both neurons and astrocytes [7]. Insulin signaling acts as a neuromodulator that regulates the release and reuptake of neurotransmitters, such as acetylcholine and norepinephrine in rat locus coeruleus [8], and regulates neuronal and glial functions such as synaptogenesis and synaptic plasticity via energy homeostasis, gene expression, and cognition [9]. Interestingly, insulin administered by intranasal improved cognitive dysfunction and insulin signaling, reduced amyloid-*β* (A*β*) production and amyloid plaque burden, and increased neurogenesis in 4-month-old APP/PS1 mice showing early Alzheimer's disease (AD) pathologies [10]. Furthermore, intranasal insulin application improved cognitive performance in healthy subjects, aged subjects, AD patients, and experimental models of insulin resistance [11]. In the brain, insulin-degrading enzyme (IDE) is synthesized and secreted by neurons, oligodendrocytes, and microglia [12]. IDE degrades extracellular A*β* in microglial and neuronal cultures and insulin can prevent this degradation, thereby impairing the clearance of extracellular A*β* [13]. IDE mutant rats, which show reduced activity of the enzyme, lead to type 2 diabetes, resulting in the enhanced cerebral deposition of A*β* [14]. However, to clarify the precise mechanisms involved in the development of diabetes-induced dementia, further research will be required.

Streptozotocin (STZ), a glucosamine-nitrosourea compound, is a genotoxic methylating agent and preferentially destroys insulin-producing *β* cells of the pancreas through the generation of ROS and alkylation of DNA [15]. STZ is a chemical used for the generation of diabetes phenotypes in most strains of rodents [16]. A mouse model utilizing five doses of STZ at low dose (50–60 mg/kg/day) has been extensively used in studies of type 1 diabetes (T1D) due to the progressive destruction of pancreatic *β* cells induced [17]. Importantly, the STZ-induced diabetes of mouse resulted in spatial learning deficits and impaired hippocampal long-term potentiation (LTP), which is thought to affect the cellular mechanisms of learning and memory [18]. Accordingly, STZ-treated mice have been used extensively to examine the physiological and pathophysiological consequences of diabetes-induced dementia.

In the present study, changes in the expression of hippocampal and cortical proteins and phosphoproteins in STZ-treated mice were examined using two-dimensional gel electrophoresis followed by staining with SYPRO Ruby and Pro-Q Diamond, respectively, and subsequent mass spectrometry to elucidate the molecular mechanisms involved in diabetes-induced dementia.

## 2. Materials and Methods

### 2.1. Reagents

Streptozotocin (STZ), urea, thiourea, sodium dodecyl sulfate (SDS), 3-((3-cholamidopropyl) dimethylammonio)-1-propanesulfonate (CHAPS), 2-mercaptoethanol (2-ME), dithiothreitol (DTT), bromophenol blue, iodoacetamide, RNase A, and DNase I were purchased from Wako Pure Chemical Industries, Ltd. (Osaka, Japan). Source information for all other assay reagents and materials is stated in the Materials and Methods section described below.

### 2.2. Animals

C57BL/6 mice (Japan SLC, Inc., Shizuoka, Japan) were maintained in a standard 12 h light/dark environment (lights on at 7:00 A.M.). Food and water were available to mice ad libitum. All experimental procedures were performed in accordance with the National Institutes of Health Guidelines on the Care and Use of Animals and confirmed by the Himeji Dokkyo University Animal Experiment Committee. All efforts were made to minimize animal use and suffering.

### 2.3. Generation of Diabetic Model Mice

The diabetic model was set up by intraperitoneal injection of STZ 50 mg/kg once a day for 5 consecutive days [19]. Ten weeks after injection, the mice were tested for sufficient levels of hyperglycemia. Blood glucose level was assessed using blood glucometer (Terumo Co. Tokyo, Japan) by tail vein puncture blood sampling. A serum glucose level higher than 400 mg/dl was considered diabetic [20].

### 2.4. Protein Extraction

Protein extraction was performed as previously described [21]. Both the STZ- and vehicle-treated mice were killed under anesthesia with pentobarbital sodium. The hippocampi and cortices were isolated from three mice in each group, and mixed separately for the two groups. Two-DE analysis was repeated in triplicate. In brief, mouse hippocampal samples were homogenized in lysis buffer [7 M urea, 2 M thiourea, 5% CHAPS, 2% immobilized pH gradient (IPG) buffer (GE Healthcare UK Ltd., Buckinghamshire, UK), 50 mM 2-ME, 2.5 *μ*g/ml DNase I, and 2.5 *μ*g/ml RNase A]. Solubilized extracts were centrifuged at 15,000 ×g for 30 min, and the supernatant was used for further analysis.

### 2.5. Two-DE

Two-DE analysis was carried out as previously described [21, 22]. In brief, one-dimensional isoelectric focusing (IEF) gel electrophoresis was performed using IPG gel strips (pH 4–7; 7 cm; GE Healthcare, WI). Approximately 1000 *μ*g of protein from each group was incubated with the IPG strips and run at 50 V for 6 h, at 100 V for 6 h, and finally at 2000 V for 6 h. After IEF gel electrophoresis, the IPG strips were equilibrated for 15 min in equilibration buffer [50 mM Tris-HCl, pH 8.8, 6 M urea, 1% SDS, 30% (v/v) glycerol, and 1% (w/v) DTT] and then for 15 min in equilibration buffer containing 2.5% (w/v) iodoacetamide instead of DTT. For the second dimension, the equilibrated IPG strips were transferred onto 15% SDS-PAGE gels at 5 mA/gel for 7 h.

### 2.6. Protein or Phosphoprotein Staining and Image Acquisition

Protein or phosphoprotein gel staining and image acquisition were carried out as previously described [21, 22]. Briefly, the gels were fixed three times in 200 ml immobilization solution [10% acetic acid and 50% methanol] for 30 min and washed five times with 200 ml of water for 15 min. Under the dark, the gels were stained with Pro-Q Diamond phosphoprotein gel stain (Life Technologies, Carlsbad, CA) for 120 min at room temperature with gentle agitation and then washed three times with destaining solution [50 mM sodium acetate, pH 4.0 and 20% (v/v) acetonitrile] for 30 min. Image acquisition was performed on Fluorophorestar 3000 image capture system (Anatech, Tokyo, Japan) with a 520 nm excitation and 575 nm emission filter for Pro-Q Diamond detection.

Next, gels were washed with washing solution [10% methanol and 7% acetic acid] for 30 min. The gels were incubated in SYPRO Ruby stain (Life Technologies) for 90 min in the dark. The gels were washed with destaining solution [10% methanol and 7% acetic acid] for 30 min and rinsed with MilliQ H_2_O. Image acquisition was carried out using a Fluorophorestar 3000 image capture system with a 470 nm excitation and 618 nm emission filter for SYPRO Ruby detection.

### 2.7. Image Analysis

Image analysis was performed as previously described [21, 22]. Image analysis and the quantification of gel spots were performed with Prodigy SameSpots software (Nonlinear Dynamics, Newcastle upon Tyne, UK). From the menu of SameSpots normalization options, we chose to normalize the intensity of each spot to the total intensity of all matched spots within each gel and to identify differentially expressed spots by comparing spot intensity differences between samples from STZ-treated mice and control mice using ANOVA.

### 2.8. Trypsin Digestion

In-gel digestion was performed using a method described [21]. Protein spots were cut from the gels, and the gel pieces were washed three times for 15 min each with 200 *μ*l of 50 mM ammonium bicarbonate with 50% (v/v) acetonitrile and then dried under vacuum. The gel piece was rehydrated in 5 *μ*l of sequencing-grade modified trypsin (10 ng/*μ*l, Promega, Madison, WI) in 10 mM ammonium bicarbonate for 30 min at 4°C, and digestion was carried out for 18 h at 37°C. Peptides were extracted with 5 *μ*l of extracting solution [50% (v/v) acetonitrile and 0.3% (v/v) trifluoroacetic acid] for 10 min by sonication.

### 2.9. Mass Spectrometry Analysis and Protein Identification

Mass spectrometry analysis was performed in accordance with the procedure described in our previous report [21]. In brief, mass spectra were obtained using a MALDI-TOF MS/MS analyzer (ABI PLUS 4800, Applied Biosystems, Foster City, CA). One *μ*l of each sample was mixed with 1 *μ*l matrix solution [1 *μ*g/*μ*l *α*-cyano-4-hydroxycinnamic acid (CHCA, Wako Pure Chemical Industries Ltd.) in 50% (v/v) acetonitrile and 0.3% (v/v) trifluoroacetic acid]. Analyte and matrix were spotted onto a stainless steel MALDI target plate and dried under ambient conditions. The peptides were analyzed using a MALDI-TOF MS/MS analyzer, and the authors searched the database with the Mascot search engine (http://www.matrixscience.com; Matrix Science, Boston, MA) using a Mascot MS/MS ion search through NCBInr databases. Proteins were considered as identified by MALDI-TOF MS if they had Mascot scores of 60 or higher (*P* < 0.05).

### 2.10. Western Blotting

The isolated hippocampus and cortex samples were homogenized in buffer containing 20 mM Tris-HCl, pH 7.0, 6 M urea, 150 mM NaCl, 2 mM EDTA, and 1% Triton X-100. The homogenates were subjected to 8% SDS-polyacrylamide gel electrophoresis and analyzed by Western blot using rabbit anti-TCTP (diluted 1 : 1000, Abcam, Cambridge, MA), rabbit anti-SUCLA2 (diluted 1 : 1000, Abcam), rabbit anti-NSE (diluted 1 : 1000, Abcam), and rabbit anti-GAPDH (diluted 1 : 10,000, AbFrontier, Seoul, Korea) antibodies at 4°C overnight. The membranes were incubated with the indicated secondary antibody (diluted 1 : 5000, GE Healthcare, Madison, WI). All values were corrected with reference to the value for GAPDH, used as an internal standard. Immunoreactivity was detected by using an Amersham ECL Prime Western blotting detection kit (GE Healthcare). Western blot images were quantified using the Multi Gauge version 2.2 software (Fuji Photofilm, Tokyo, Japan).

### 2.11. Statistical Analysis

Respective densitometric quantifications are shown as mean ± SEM. All data were tested by one-way ANOVA followed by Dunnett's multiple-comparisons tests to evaluate the differences between groups. *P* < 0.05 was considered statistically significant.

## 3. Results

**Figure 1:**
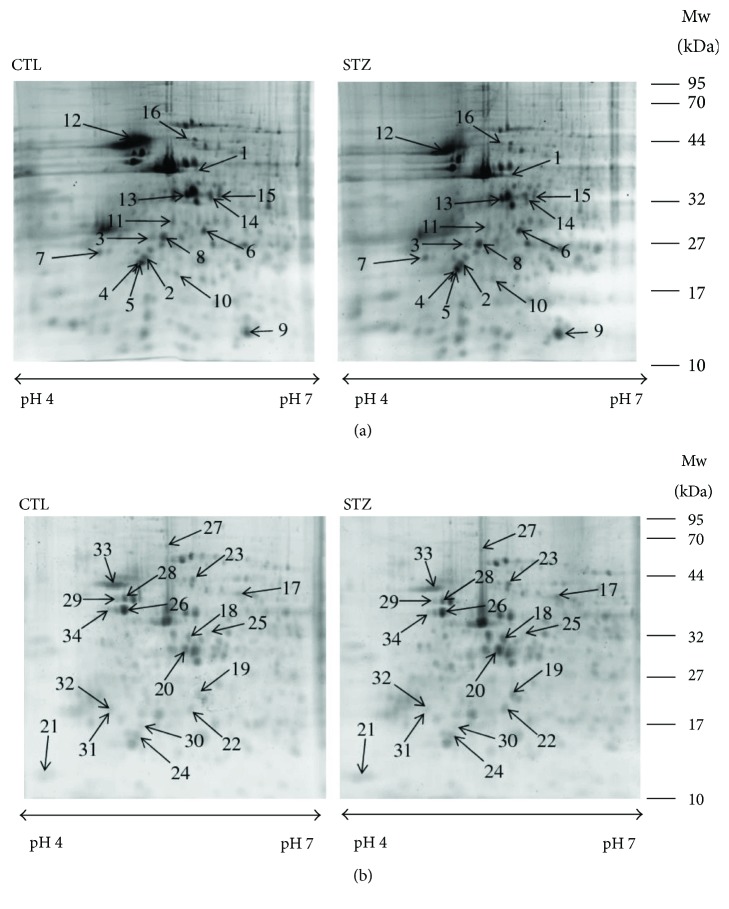
Two-DE images of hippocampal (a) and cortical (b) proteins in STZ-treated (STZ) and control (CTL) mice. IPG strips (pH 4–7) were used for the first dimension, and 15% polyacrylamide gels were used for the second dimension. Isoelectric points (PIs) and experimental masses are shown on the *x*- and *y*-axes. The differentially expressed proteins are identified by numbers that correspond to spot numbers in Tables 1 and 3. Experiments were repeated three times independently with similar results. Typical gel images are shown.

**Figure 2:**
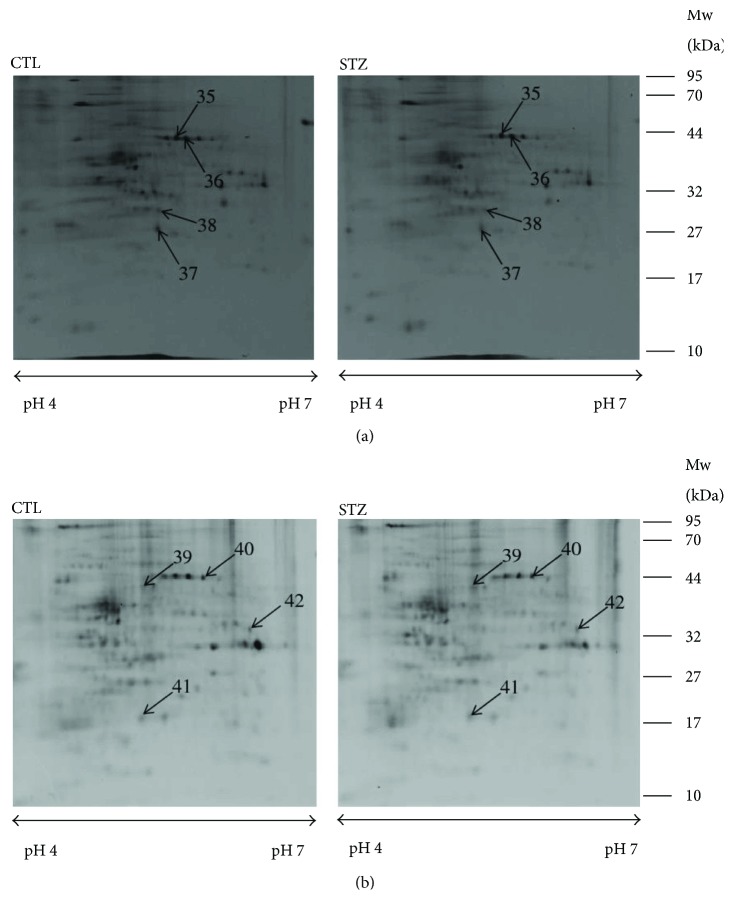
Two-DE images of hippocampal (a) and cortical (b) phosphoproteins in STZ-treated (STZ) and control (CTL) mice. IPG strips (pH 4–7) were used for the first dimension, and 15% polyacrylamide gels were used for the second dimension. Isoelectric points (PIs) and experimental masses are shown on the *x*- and *y*-axes. The differentially expressed phosphoproteins are identified by numbers that correspond to spot numbers in Table 2. Experiments were repeated three times independently with similar results. Typical gel images are shown.

The expression changes of proteins and phosphoproteins in the hippocampus and cortex of STZ-treated and untreated control mice were quantified and identified on 2-DE gels using Prodigy SameSpot software and MALDI-TOF MS/MS. Image analysis showed approximately 400 protein spots and 200 phosphoprotein spots on each SYPRO Ruby-stained 2-DE gel (Figure 1) and each Pro-Q Diamond-stained 2-DE gel (Figure 2), respectively. We detected 16 (5 up- and 11 downregulated) hippocampal proteins (Table 1), 16 (7 up- and 9 downregulated) cortical proteins (Table 2), 3 (1 up- and 2 downregulated) hippocampal phosphoproteins (Table 3), and 4 (2 up- and 2 downregulated) cortical phosphoproteins (Table 3). These proteins and phosphoproteins were categorized into functional groups as shown in Tables 1–3 using the PANTHER (http://www.pantherdb.org/) database.

### 3.1. Identification of Altered Proteins and Phosphoproteins in the Hippocampus of STZ-Treated Mice

The 5 proteins with increased expression levels were identified as type II peroxiredoxin 1, ATP-specific succinyl-CoA synthetase beta subunit, rho GDP-dissociation inhibitor 1, heme-binding protein, and phosphatidylethanolamine-binding protein 1, and the 11 proteins with decreased expression levels were identified as profilin-2, tubulin beta-5 chain, alpha-internexin, ketimine reductase mu-crystallin, L-lactate dehydrogenase B chain isoform 1, ubiquitin carboxy-terminal hydrolase L1, isoform CRA_a, proteasome subunit alpha type-3, N(G),N(G)-dimethylarginine dimethylaminohydrolase 1, ferritin heavy chain, translationally controlled tumor protein, and prohibitin (Table 1).

The phosphoprotein with increased expression level was identified as dihydropyrimidinase-related protein 2, and 2 phosphoproteins with decreased expression level were identified as proteasome subunit alpha type-3 and beta-soluble NSF attachment protein (Table 2).

### 3.2. Identification of Proteins and Phosphoproteins with Altered Expression in the Cortex of STZ-Treated Mice

The 7 proteins with increased expression levels were identified as alpha-tubulin, partial, tubulin alpha-1C chain, put. Beta-actin (aa 27–375), gamma-actin, alpha-internexin, beta-synuclein, and unnamed protein product, and the 9 proteins with decreased expression levels were identified as tubulin beta-5 chain, NADH dehydrogenase (ubiquinone) Fe-S protein 1, atp5b protein, partial, gamma-enolase isoform 1, calretinin, heme-binding protein, phosphatidylethanolamine-binding protein 1, COP9 signalosome complex subunit 4, and ubiquitin C-terminal hydrolase L3 (Table 3).

The 2 phosphoproteins with increased expression levels were identified as dihydropyrimidinase-related protein 2 and enolase 1B, retrotransposed, and the 2 phosphoproteins with decreased expression levels were identified as gamma-actin and proteasome subunit alpha type-3 (Table 2).

### 3.3. Western Blot Analysis of the Altered Proteins in the Hippocampus and Cortex of STZ-Treated Mice

**Figure 3:**
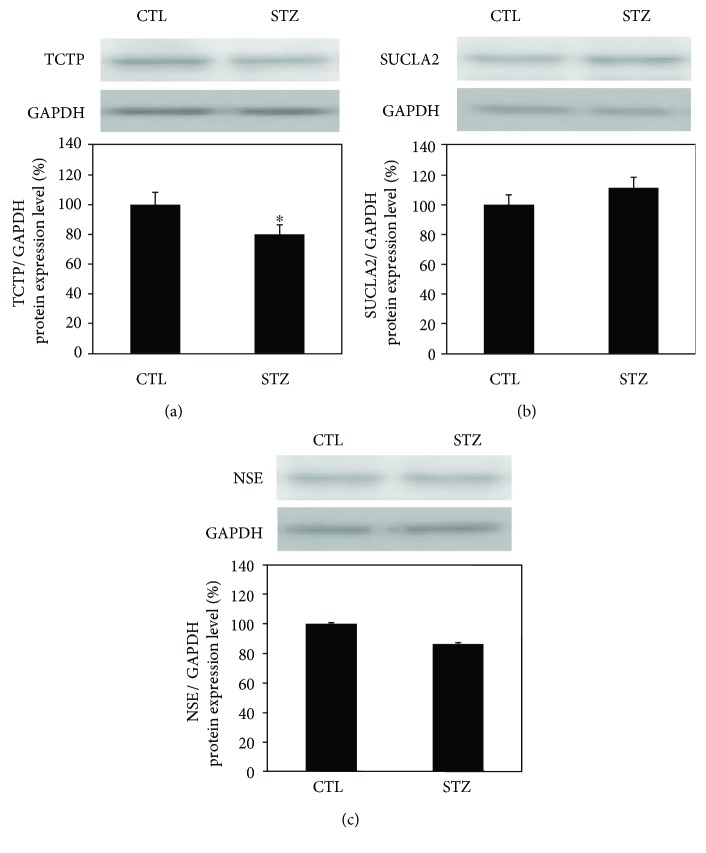
Representative images of Western blotting evaluating the expression of translationally controlled tumor protein (TCTP) (a), ATP-specific succinyl-CoA synthetase beta subunit (SUCLA2) (b) in the hippocampus, and gamma-enolase isoform 1 (NSE) (c) in the cortex from STZ-treated (STZ) and control (CTL) mice. The protein expression levels are normalized to glyceraldehyde 3-phosphate dehydrogenase (GAPDH) expression and indicated as the ratio relative to that in CTL. Data are presented as mean ± SEM of four independent experiments. Representative bands are shown above each graph. ^∗^*P* < 0.05 versus control.

Western blot analysis was performed to validate the identity of translationally controlled tumor protein and ATP-specific succinyl-CoA synthetase beta subunit as differentially expressed hippocampal proteins and the identity of gamma-enolase isoform 1 as differentially expressed cortical protein. The protein level of translationally controlled tumor protein was significantly decreased about 0.8-fold in the hippocampus of STZ-treated mice compared with untreated control (*P* = 0.049) (Figure 3(a)). The protein level of hippocampal ATP-specific succinyl-CoA synthetase beta subunit tended to increase (*P* = 0.29) (Figure 3(b)) and that of cortical gamma-enolase isoform 1 tended to decrease (*P* = 0.057) (Figure 3(c)).

## 4. Discussion

In this study, we used 2-DE coupled with MS to investigate changes in the expression of proteins and phosphoproteins in the hippocampus and cortex of STZ-treated mice, that is, diabetic model mice showing dementia, and revealed that the expression of 32 proteins and 7 phosphoproteins changed significantly.

### 4.1. Cellular Cytoskeleton

Micorotubules are composed of *α*- and *β*-tubulin heterodimers and are present throughout neuronal dendrites and axons. Microtubule dynamics regulate axonal outgrowth, dendritic spine morphology, and synaptic plasticity [23]. Treatment with paclitaxel, a microtubule dynamics inhibitor, leads to LTP deficits in the cortico-amygdala pathway in mouse brain slices [24]. Actin exists in both monomeric (G-actin) and polymerized (F-actin) forms and presents in dendritic spines. Actin dynamics are essential in synaptic function and memory formation [25]. Indeed, cytochalasin D, an inhibitor of F-actin polymerization, blocks the late phase of LTP but not the early phase [26]. Thus, changes in the expression of tubulin and actin might affect synaptic plasticity, being involved with diabetes-induced dementia.

### 4.2. Oxidoreductase

Peroxiredoxins (Prxs) are antioxidant enzymes that contain one or two cysteine (Cys) residues in their active site [27]. There are six isoforms divided into three groups: the 2-Cys Prxs (Prx 1, 2, 3, and 4), the atypical 2-Cys Prx (Prx 5), and the 1-Cys Prx (Prx 6) [27]. In neurons, intracellular Prxs, which are induced by various oxidative stimuli, protect against oxidative radical damage by ROS [28]. Type II Prx 1, also known as Prx 2, is predominantly expressed in the brain [29]. The protein level of Prx 2 is increased in aging and AD brains, suggesting that Prx 2 is involved in the elevated neuronal antioxidant response under oxidative stress [30]. The proteomic analysis of the hippocampus in AD patients shows that the expression of Prx 2 increases compared with age-matched controls [31]. Prx 2-deficient mice show remarkably increased susceptibility to oxidative stress-induced tissue damage [32]. Therefore, the increased expression of Prx 2 might decrease oxidative damage, improving abnormalities of neurons and glia in the brain and subsequent dementia.

### 4.3. Neurotransmitter Release

Translationally controlled tumor protein (TCTP) is a multifunctional protein that is involved in immune responses, cell proliferation, cancer progression, and apoptosis [33]. TCTP, also known as histamine-releasing factor (HRF), induces the secretion of histamine that is widely distributed in the brain [34]. Histamine-expressing neurons project to wide areas of the brain, including regions especially important for cognitive functions such as the frontal cortex and hippocampus [35]. Histamine and TCTP are significantly reduced in AD brain compared to age-matched control, suggesting that decreased histamine levels impair cognitive function in AD [35]. Thus, the decreased expression of TCTP might decrease the histamine release and be one of the main causes of dementia.

### 4.4. Protein Deubiquitination

In humans, four ubiquitin carboxy-terminal hydrolase (UCH) proteins (UCH-L1, UCH-L3, UCH37/UCH-L5, and BAP1) have been identified, but only UCH-L1 and L3 have been studied in detail [36]. UCH-L1 is a neuronal deubiquitinase that cleaves peptide adducts from the C-terminus of ubiquitin [37]. UCH-L1 is predominantly expressed in the brain [37]. The proteomic analysis of the hippocampus in A*β*PPswe/PS1dE9 mice shows that the expression of UCH-L1 decreases compared with age-matched wild-type mice [38]. UCH-L1 is downregulated in the brain of patients with Parkinson's disease and AD [39]. The administration of UCH-L1 protein fused to the transduction domain of HIV transactivator (TAT) protein into APP/PS1 model mice of AD provided a protective effect against amyloid-induced neurodegeneration in synaptic function and contextual memory [40]. UCH-L3 is universally expressed in all tissues [41]. UCH-L3-deficient mice exhibit significant impairment in learning and memory using Morris water maze and 8-arm radial maze task [42]. Indeed, our findings showed that UCH-L1 and L3 were downregulated in diabetes-induced dementia. Thus, the decreased expressions of UCH-L1 and L3 could contribute to neurodegeneration, resulting in dementia.

### 4.5. Antiapoptosis

The human synuclein family has 3 members, *α*-synuclein, *β*-synuclein, and *γ*-synuclein [43]. *α*-Synuclein and *β*-synuclein are predominantly localized at presynaptic nerve terminals in the CNS [44]. In contrast, *γ*-synuclein is abundant in the peripheral nervous system [45]. *β*-Synuclein protects neurons against apoptosis induced by staurosporine and 6-hydroxydopamine, which is linked to the suppression of p53 transcriptional activity [46]. Thus, the increased expression of *β*-synuclein could protect against neuronal apoptosis in diabetes-induced dementia.

### 4.6. Calcium Ion Binding

Calretinin is an EF-hand calcium-binding protein involved in calcium signaling [47]. Calretinin is expressed in hilar mossy cells and in widely distributed subsets of GABAergic interneurons in the normal mouse hippocampus [48]. Calretinin maintains appropriate calcium ion concentration in cells and participates in the modulation of neuronal activity and synaptic plasticity [49]. Knockout mice lacking calretinin show no alteration in basal synaptic transmission but impaired LTP in the dentate gyrus [48]. These findings indicate that the decreased expression of calretinin might be involved in diabetes-induced dementia.

### 4.7. Neuronal Development

Dihydropyrimidinase-related protein 2/collapsin response mediator protein 2 (DPYL2/CRMP2) binds to tubulin heterodimers to promote microtubule formation and stability, resulting in axonal growth and neuronal polarity [50]. Cyclin-dependent kinase 5 (Cdk5) and glycogen syntheses kinase 3*β* (GSK-3*β*) regulate DPYL2 activity [51]. The phosphorylation of DPYL2 by GSK-3*β* can inactivate DPYL2 function [52]. DPYL2 is phosphorylated at Ser522 by Cdk5 and subsequently at Ser518, Thr514, and Thr509 by GSK-3*β* in brain tissue from human AD patients and in some mouse models of AD [53]. A*β*_25–35_-induced impairment of cognitive function and LTP was not observed in DPYL2 phosphorylation-deficient knock-in mice, in which Ser522 of DPYL2 was replaced with alanine [54], suggesting that the phosphorylation of DPYL2 at Ser522 was associated with A*β*_25–35_-induced cognitive memory deficit and impairment of LTP. Indeed, our findings showed that phosphorylated DPYL2 was increased in the hippocampus and cortex of STZ-treated mice. Taken together, it is suggested that phosphorylated DPYL2 plays an important role in diabetes-induced dementia. Interestingly, DPYL2 was identified from two phosphoprotein spots on 2-DE gels stained with Pro-Q Diamond staining in the hippocampus. The shift in the position of these phosphoprotein spots may imply translational modifications such as phosphorylation, acetylation, and degradation.

## 5. Conclusion

In conclusion, we found 32 proteins and 7 phosphoproteins with significantly altered levels in the hippocampus and cortex of STZ-treated mice. We propose that the identified proteins and phosphoproteins might play important roles in the molecular mechanisms involved in diabetes-induced dementia.

## Figures and Tables

**Table 1:** Identified proteins in the hippocampus and their functional categories.

Spot no.	Accession no.	Identified protein	Observed Mw (kDa)/pI	Theoretical Mw (Da)/pI	Score	SC	Fold change (STZ/CTL)	ANOVA *P* value
*Cytoskeletal protein*									
9	gi|9506971	Profilin-2	12/6.2	15,364/6.55	145	28	0.8	0.048
12	gi|7106439	Tubulin beta-5 chain	43/5.0	50,095/4.78	295	14	0.8	0.012
16	gi|508538	Alpha-internexin	43/5.6	56,007/5.16	232	14	0.7	0.013
*Oxidoreductase*									
5	gi|3603241	Type II peroxiredoxin 1	21/5.1	21,949/5.20	136	9	1.1	0.032
13	gi|7710012	Ketimine reductase mu-crystallin	32/5.6	33,673/5.44	208	14	0.8	0.002
15	gi|6678674	L-Lactate dehydrogenase B chain isoform 1	32/5.9	36,834/5.70	233	14	0.7	0.041
*Protein deubiquitination*									
8	gi|148705825	Ubiquitin carboxy-terminal hydrolase L1, isoform CRA_a	26/5.3	24,977/5.05	221	21	0.9	0.018
11	gi|261824000	Proteasome subunit alpha type-3	28/5.4	28,615/5.29	201	17	0.8	0.043
*Energy metabolism*									
1	gi|3766201	ATP-specific succinyl-CoA synthetase beta subunit	37/5.7	46,557/5.65	170	8	1.8	0.014
*GTPase activation*									
3	gi|31982030	Rho GDP-dissociation inhibitor 1	26/5.2	23,450/5.12	114	15	1.2	0.038
*Heme binding*									
2	gi|3724328	Heme-binding protein	22/5.1	21,165/5.18	95	14	1.3	0.023
*Hydrolase*									
14	gi|38371755	N(G),N(G)-dimethylarginine dimethylaminohydrolase 1	31/5.8	31/5.8 31,760/5.64	125	8	0.7	0.011
*Iron storage*									
10	gi|6753912	Ferritin heavy chain	18/5.5	21,224/5.53	237	26	0.8	0.039
*Neurotransmitter release*									
7	gi|6678437	Translationally controlled tumor protein	24/4.7	19,564/4.76	98	5	0.9	0.015
*Protease inhibitor*									
4	gi|84794552	Phosphatidylethanolamine-binding protein 1	21/5.1	20,988/5.19	168	25	1.1	0.032
*Transcription*									
6	gi|6679299	Prohibitin	27/5.7	29,859/5.57	270	20	0.9	0.038

Spot numbers correspond to the 2-DE gel in Figure 1(a). Accession numbers were obtained from the National Center for Biotechnology Information (NCBI) database. Score and sequence coverage (SC) were obtained by Mascot database searching (http://www.matrixscience.com). *P* values were obtained by ANOVA, *P* < 0.05.

**Table 2:** Identified proteins in the cortex and their functional categories.

Spot no.	Accession no.	Identified protein	Observed Mw (kDa)/pI	Theoretical Mw (Da)/pI	Score	SC	Fold change (STZ/CTL)	ANOVA *P* value
*Cytoskeletal protein*									
17	gi|3642627	Alpha-tubulin, partial	40/6.1	11,058/4.85	70	15	1.4	0.016
18	gi|6678469	Tubulin alpha-1C chain	31/5.6	50,562/4.96	187	10	1.4	0.015
19	gi|49868	Put. beta-actin (aa 27–375)	25/5.7	39,446/5.78	126	8	1.3	0.035
22	gi|809561	Gamma-actin	22/5.6	41,335/5.56	221	10	1.3	0.037
23	gi|148539957	Alpha-internexin	44/5.6	55,520/5.35	567	19	1.2	0.045
33	gi|7106439	Tubulin beta-5 chain	41/4.9	50,095/4.78	426	12	0.7	0.031
*Energy metabolism*									
27	gi|13879366	NADH dehydrogenase (ubiquinone) Fe-S protein 1	59/5.4	80,724/5.51	264	10	0.9	0.002
28	gi|23272966	Atp5b protein, partial	39/5.0	56,632/5.24	535	22	0.8	0.007
29	gi|23272966	Atp5b protein, partial	39/4.9	56,632/5.24	470	19	0.8	0.008
*Glycolysis*									
26	gi|7305027	Gamma-enolase isoform 1	36/5.0	47,609/4.99	449	14	0.9	0.044
34	gi|7305027	Gamma-enolase isoform 1	36/4.8	47,609/4.99	249	11	0.6	0.004
*Antiapoptosis*									
21	gi|15809030	Beta-synuclein	16/4.2	14,043/4.38	122	29	1.3	0.024
*Calcium ion binding*									
31	gi|34098931	Calretinin	22/4.8	31,467/4.94	199	15	0.8	0.008
*Heme metabolic process*									
30	gi|4886904	Heme-binding protein	20/5.2	21,153/5.18	170	26	0.8	0.004
*Protease inhibitor*									
24	gi|84794552	Phosphatidylethanolamine-binding protein 1	19/5.1	20,988/5.19	203	32	0.9	0.007
*Protein degradation*									
25	gi|6753490	COP9 signalosome complex subunit 4	31/5.8	46,541/5.57	62	4	0.9	0.035
*Protein deubiquitination*									
32	gi|7578956	Ubiquitin C-terminal hydrolase L3	22/4.8	26,333/5.08	113	9	0.8	0.008
*Unknown*									
20	gi|74212109	Unnamed protein product	29/5.5	50,076/4.75	246	10	1.3	0.041

Spot numbers correspond to the 2-DE gel in Figure 1(b). Accession numbers were obtained from the National Center for Biotechnology Information (NCBI) database. Score and sequence coverage (SC) were obtained by Mascot database searching (http://www.matrixscience.com). *P* values were obtained by ANOVA, *P* < 0.05.

**Table 3:** Identified phosphoproteins in the hippocampus and cortex, and their functional categories.

Spot no.	Accession no.	Identified protein	Observed Mw (kDa)/pI	Theoretical Mw (Da)/pI	Score	SC	Fold change (STZ/CTL)	ANOVA *P* value
Hippocampus									

*Neuronal development*									
35	gi|40254595	Dihydropyrimidinase-related protein 2	44/5.6	62,638/5.95	85	4	1.2	0.04
36	gi|40254595	Dihydropyrimidinase-related protein 2	44/5.8	62,638/5.95	144	6	1.1	0.015
*Protein degradation*									
37	gi|261824000	Proteasome subunit alpha type-3	28/5.3	28,615/5.29	88	9	0.9	0.037
*Vesicular transport*									
38	gi|29789104	Beta-soluble NSF attachment protein	30/5.3	33,878/5.32	177	16	0.8	0.04

Cortex									

*Cytoskeletal protein*									
42	gi|809561	Gamma-actin	43/5.3	41,335/5.56	254	13	0.7	0.039
*Neuronal development*									
39	gi|40254595	Dihydropyrimidinase-related protein 2	45/5.9	62,638/5.95	490	17	1.3	0.026
*Protein degradation*									
41	gi|261824000	Proteasome subunit alpha type-3	21/5.3	28,615/5.29	142	14	0.8	0.043
*Unknown*									
40	gi|70794816	Enolase 1B, retrotransposed	32/6.5	47,453/6.37	163	9	1.1	0.018

Spot numbers correspond to the 2-DE gel in Figure 2. Accession numbers were obtained from the National Center for Biotechnology Information. (NCBI) database. Score and sequence coverage (SC) were obtained by Mascot database searching (http://www.matrixscience.com). *P* values were obtained by ANOVA, *P* < 0.05.
